# 

**Published:** 1886-12

**Authors:** 


					\ ?
V DECEMBER, 1886. 1/VvA ! / T>- . ..
Vol. IVI
til ? 3== ? No. 14.
I snoicra:-: the y?^Jbra^>
II ' INDEX F
" BRISTOL
M EDICC)-C111RURGICAL
JOURNAL
% (SluarteriB Journal of tbc /ifce&fcal Sciences for tbe IRaest
of England ant) Soutb TKHales
PUBLISHED UNDER THE AUSPICES OF
THE BRISTOL MEDICO-CHIRURGICJL SOCIETT.
EDITOR :
J. G R E I G SMITH,
M.A., F.R.S.E.
ASSISTANT-EDITOR :
L. M. GRIFFITHS.
"Sckc cet ncscfre, nfef fo me
Scire alius sdret."
BRISTOL: J. W. ARROWSMITH;
London: J. & A. Churchill, 11 New Burlington Street.

				

